# Supplementary material for: Paired Primary and Recurrent Rhabdoid Meningiomas: Cytogenetic Alterations, BAP1 Gene Expression Profile and Patient Outcome
Source: Biology (Basel). 2024 May 16;13(5):350. doi: 10.3390/biology13050350 (PMC11117813; doi:10.3390/biology13050350)

Table S1. Clinical features of RM patients.

ID sample	Year of diagnosis	Sex	Age	WHO grade	N. of recurrences	Type of surgery	RDT	Alive status	Follow up (years)
P1_p	1998	Male	51	2	1	STR	No	Alive	22
P1_R1	2006		59	3		GTR	No		
P2_p	2010	Male	68	3	1	GTR	Yes	Alive	13
P2_R1	2017		75	3		GTR	Yes		
P3_p	2016	Male	58	2	2	GTR	Yes	Alive	7
P3_R1	2021		63	3		GTR	Yes		
P3_R2[&]	2023		65	-		No	No		
P4_p	1997	Female	45	1	2	GTR	No	Alive	13
P4_R1	2000		48	3		GTR	Yes	Alive	
P4_R2	2010		58	3		GTR	No	Dead	
P5_p	2007	Male	65	2	3	GTR	No	Alive	16
P5_R1	2011		69	3		GTR	No	Alive	
P5_R2	2014*		72	3		STR	Yes	Alive	
P5_R3	2020		78	3		STR	Yes	Alive	
P6_p[ϴ]	1998	Female	61	2	3	GTR	No	Alive	15
P6_R1	2007		70	3		GTR	No	Alive	
P6_R2	2009		72	3		STR	Yes	Alive	
P6_R3	2010		73	3		RadioS	Yes	Dead	
P7_p	2007	Female	69	1	3	GTR	No	Alive	11
P7_R1	2009		71	2		GTR	No	Alive	
P7_R2	2012		74	2		GTR	Yes	Alive	
P7_R3	2015**		79	3		STR	Yes	Dead	
P8	2011	Male	34	3	0	GTR	Yes	Alive	11
P9	2011	Male	65	3	0	GTR	No	Alive	11
P10	2011	Male	65	3	0	GTR	No	Dead***	-
P11	2018	Female	50	3	0	GTR	No	Alive	6
P12	2017	Male	54	3	0	GTR	Yes	Dead	3
P13	2018	Male	53	3	0	STR	Yes	Alive	6
P14	2019	Male	81	3	0	GTR	Yes	Alive	4
P15	2001	Female	72	3	0	GTR	No	Loss	-

p: primary tumor sample; R: recurrence samples label as 1 for first relapse, 2 for the second one and R3 for the third recurrence; &: second relapse detected recently via magnetic resonance imaging (MRI); ϴ: primary calcified tumor excluded by low quality; *: a second recurrence was detected three years after the first relapse and the P5 received RDT and delayed surgical resection for another four years; **: a third recurrence was detected in 2015 and its surgical resection was done in 2017; ***: perioperative death; STR: subtotal resection; GTR: gross total resection; RadioS: radiosurgery

Table S2. Different genetic changes observed in paired primary and recurrent tumors.

Group of alteration	Type of change	Involved chromosomes		Patient (recurrent specimen)
		Previous chr	New chr	
Same chr	Increased size	del2q	gain2q	P4 (R1)
		-10q	-10	P5 (R3)
		del10p	gain10p	P4 (R2)
		del19p	gain19p	P4 (R2)
		del22q	-22$^{\&}$/ gain22q$^{\$}$	P3$^{\&}$ (R1) / P4$^{\$}$ (R2)
		-X	gainXq	P6 (R2)
	Decreased size	-8	-8p	P5 (R1)
		-11	LOH11	P6 (R2)
		-19p	del19p	P5 (R1)
	Disappear &appear	-10, -11, -X	-10, -11, -X	P6 (R2 to R3)
Different chromosomal arm	Gain	del1p	+1q	P4 (R1) / P6 (R2)
		-2p	gain2q	P4 (R1) / P6 (R2&R3)
		del6q	gain6p	P4 (R1)
		del10p	gain10q	P4 (R2)
		del19p	gain19pq	P4 (R1)
		del22q	gain22q	P4 (R2)
	Loss	2q	-2p	P4 (R2)
		del10q	del10p	P4 (R1)
Different chr	Gain (small gain or trisomy)	-	gain6p$^{\$}$, +8$^{\$}$, gain10p$^{\$,}$ +16p$^{\$}$, gain19pq$^{\$}$, +20$^{\$}$	P4$^{\$}$ (R1&R2)
			+5p$^{\$\$}$, gain7q$^{\$\$}$, gain22q$^{\$\$}$	P4$^{\$\$}$ (R2)
	Loss (deletion or monosomy)	-	-3p$^{\$}$, -4q$^{\$}$, del11p$^{\$}$, del11q$^{\$}$, del16q$^{\$}$, -X$^{\$}$	P4$^{\$}$ (R1&R2)
			-5q$^{\$\$}$, del15q$^{\$\$}$, del16p$^{\$\$}$	P4$^{\$\$}$ (R2)
			del4p*, del4q*, del7pq*, del8*, del9p*,-10q*, del12q*, 20*, 22*	P7* (R3)
			del6q*$^{\&}$	P3$^{\&}$ (R1) / P7* (R3)
	LOH	-	3q	P4 (R1)

Chr: chromosome; del: deletion; -: monosomy; P: patient; R1: first recurrent tumor; R2: second recurrent tumor; R3: third recurrent tumor; +: trisomy; LOH: loss of heterozigosity; $^{\&}$ abnormality presents in R1 of P3; $^{\$}$ abnormality presents in R1 and R2 of P4; $^{\$\$}$ abnormality presents in R2 of P4; * abnormality presents in R3 of P7.

Figure S1: Identical copy number alterations found in primary vs. recurrent RM specimens of two patients.

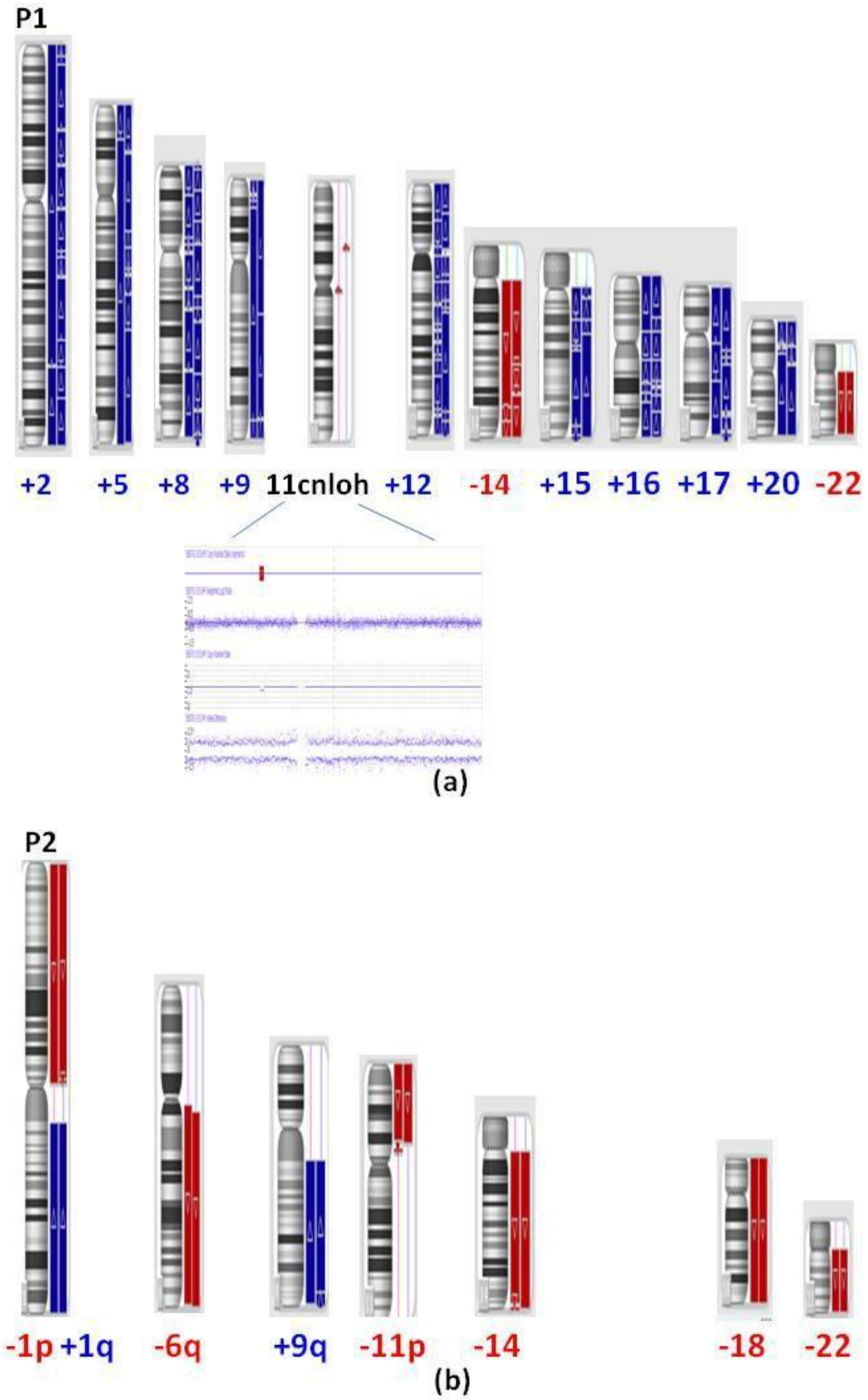

Figure S2: Chromosome losses and gains from diagnostic (primary tumors, P) to tumor recurrent specimens (labeled from the first to the third recurrence as R1, R2 and R3) obtained in 5 patients (a) patient 3 (P3), (b) patient 5 (P5), (c) patient 6 (P6), (d) patient 7 (P7) and (e) patient 4 (P4). Please note that all cases had additional genetic changes in follow-up vs. diagnostic samples. Genetic losses are highlighted in green while gains are highlighted in red. Green lines show small-size losses in the same chromosome, black lines show losses of larger regions, while red lines show new gains in the same chromosome and blue lines indicate the absence of a previous genetic abnormality and its emergence in a subsequent (third) recurrent tumor involving chromosomes 1, 11 and X. Genetic abnormalities surrounded by a blue oval line/box indicate alterations shared in 2 relapses

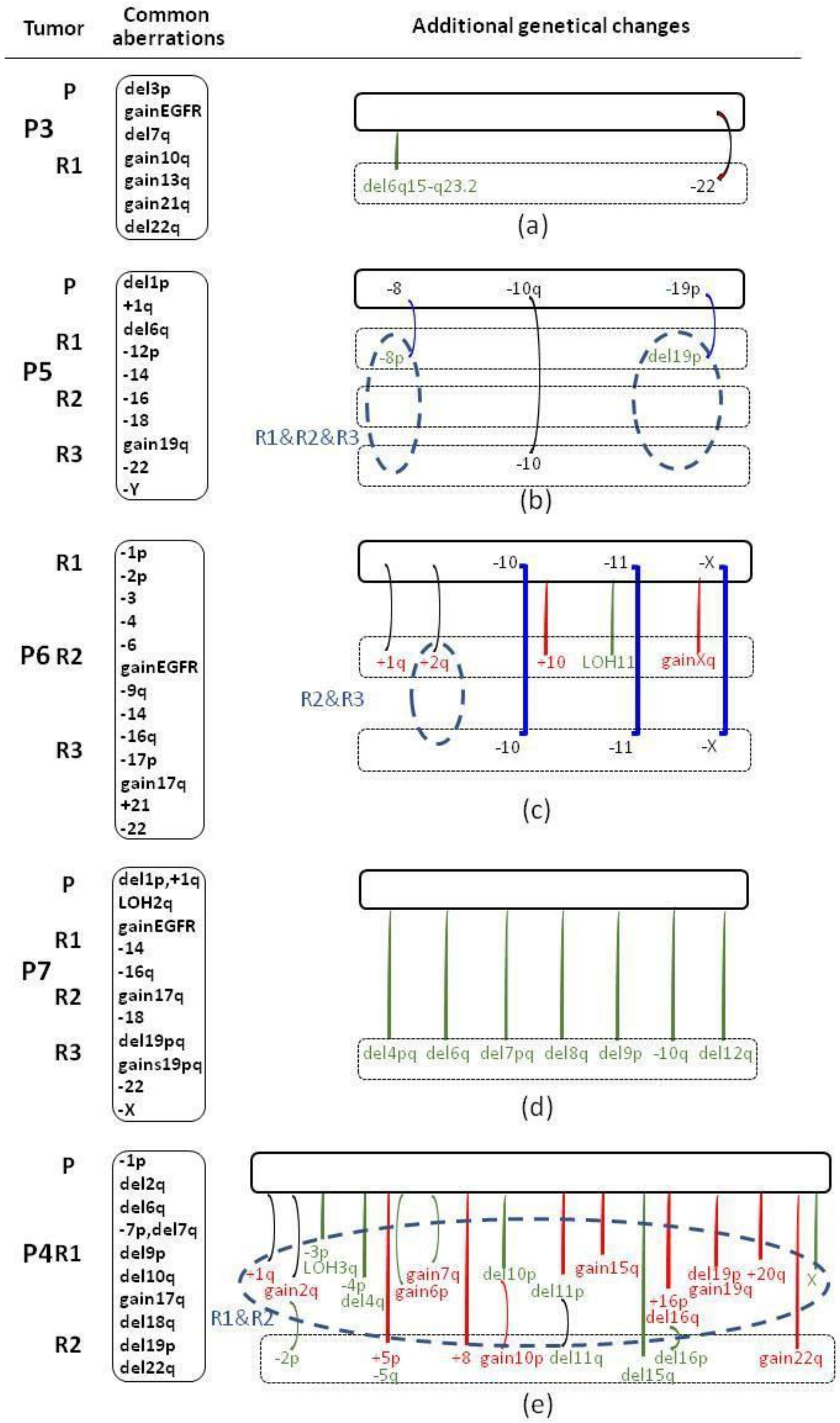

Supplement: Supplementary file 1 [file biology-13-00350-s001.zip › biology-2952870-supplementary.pdf]
